# Discrepancies in tumor mutation burden reporting from sequential endobronchial ultrasound transbronchial needle aspiration samples within single lymph node stations - brief report

**DOI:** 10.3389/fonc.2023.1259882

**Published:** 2023-10-19

**Authors:** David Fielding, Andrew J. Dalley, Mahendra Singh, Lakshmy Nandakumar, Vanessa Lakis, Haarika Chittoory, David Fairbairn, Ann-Marie Patch, Stephen H. Kazakoff, Kaltin Ferguson, Farzad Bashirzadeh, Michael Bint, Carl Pahoff, Jung Hwa Son, Kimberley Ryan, Alan Hodgson, Sowmya Sharma, John V. Pearson, Nicola Waddell, Sunil R. Lakhani, Gunter Hartel, Peter T. Simpson, Katia Nones

**Affiliations:** ^1^ Department of Thoracic Medicine, The Royal Brisbane & Women’s Hospital, Brisbane, QLD, Australia; ^2^ UQ Centre for Clinical Research, Faculty of Medicine, The University of Queensland, Brisbane, QLD, Australia; ^3^ Pathology Queensland, The Royal Brisbane & Women’s Hospital, Brisbane, QLD, Australia; ^4^ QIMR Berghofer Medical Research Institute, Brisbane, QLD, Australia; ^5^ Department of Thoracic Medicine, Sunshine Coast University Hospital, Birtinya, QLD, Australia; ^6^ Department of Respiratory Medicine, Gold Coast University Hospital, Southport, QLD, Australia; ^7^ ACL Pathology, Sydney, NSW, Australia; ^8^ School of Biomedical Sciences, The University of Queensland, Brisbane, QLD, Australia

**Keywords:** endobronchial ultrasound-guided transbronchial needle aspiration (EBUS TBNA), lung cancer, cytology, TSO500, molecular diagnostics, tumor mutation burden

## Abstract

**Introduction:**

Tumour Mutation Burden (TMB) is a potential biomarker for immune cancer therapies. Here we investigated parameters that might affect TMB using duplicate cytology smears obtained from endobronchial ultrasound transbronchial needle aspiration (EBUS TBNA)-sampled malignant lymph nodes.

**Methods:**

Individual Diff-Quik cytology smears were prepared for each needle pass. DNA extracted from each smear underwent sequencing using large gene panel (TruSight Oncology 500 (TSO500 - Illumina)). TMB was estimated using the TSO500 Local App v. 2.0 (Illumina).

**Results:**

Twenty patients had two or more Diff-Quik smears (total 45 smears) which passed sequencing quality control. Average smear TMB was 8.7 ± 5.0 mutations per megabase (Mb). Sixteen of the 20 patients had paired samples with minimal differences in TMB score (average difference 1.3 ± 0.85). Paired samples from 13 patients had concordant TMB (scores below or above a threshold of 10 mutations/Mb). Markedly discrepant TMB was observed in four cases, with an average difference of 11.3 ± 2.7 mutations/Mb. Factors affecting TMB calling included sample tumour content, the amount of DNA used in sequencing, and bone fide heterogeneity of node tumour between paired samples.

**Conclusion:**

TMB assessment is feasible from EBUS-TBNA smears from a single needle pass. Repeated samples of a lymph node station have minimal variation in TMB in most cases. However, this novel data shows how tumour content and minor change in site of node sampling can impact TMB. Further study is needed on whether all node aspirates should be combined in 1 sample, or whether testing independent nodes using smears is needed.

## Introduction

Tumour Mutation Burden (TMB) has been reported to correlate with response to checkpoint inhibitor therapy in lung cancer patients (1, 2). TMB measures the number of somatic mutations per megabase (Mb) of the genome sequenced; the score was initially assessed using Whole Exome Sequencing however comprehensive panels are increasingly being used to explore TMB (3). Tumour heterogeneity occurs in lung cancer specimens whereby different portions of a tumour are comprised of cancer cells with different genomic mutation profiles (4). This tumour heterogeneity has recently been shown to impact disease free survival (4) and may also impact the results of Programmed Cell Death Ligand (PD-L1) immunohistochemistry and TMB estimation of samples taken from different parts of a primary tumour, or when assessing regional metastatic lymph nodes. A recent report analysed the potential of tumour heterogeneity on TMB estimation using whole genome sequencing and a smaller targeted gene sequencing panel (5). Overall, TMB scores were similar between primary and metastatic lymph nodes, however, in three of 10 cases the paired samples had discrepant TMB being on either side of the threshold of 10 mutations/Mb. The results were important because they demonstrated the feasibility of TMB analysis on frozen EBUS TBNA aspirates, an extremely common way that advanced lung cancers are diagnosed; and provided the overall reassurance that the TMB results were generally reliable. However, the work also highlighted the need to be cognisant of the challenges of TMB assessment as reportable data might be impacted by tumour heterogeneity or other sample related factors such as tumour content, DNA input, and amount of immune or stroma cells.

Recently we studied the feasibility of using Diff-Quik cytology smears routinely produced during the EBUS TBNA procedure as an alternative source of tumour material for genomic testing (6). We used EBUS TBNA aspirates deposited on Diff-Quik stained cytology smears from individual passes into mediastinal nodes as a way to directly compare tumour content and DNA yield and the subsequent success of sequencing using the TSO500 assay for identifying actionable mutations (7). The sequencing of multiple smears per EBUS TBNA procedure enabled us to further explore the issue of reliability of these specimens for TMB assessment. We observed a similar level of concordance and discordance in TMB assessment between replicate patient samples as others recently reported (5), and here we explore the reasons for the discrepancy in TMB values, and discuss the implications of these on EBUS TBNA sampling best practice.

## Methods

An Institutional Review Board approved study was undertaken (HREC/17/QRBW/301; The University of Queensland (2005000785); QIMR – P2404) and patients gave written informed consent for the use of their samples for research. No results were returned to patients. Inclusion criteria was patients undergoing EBUS TBNA for diagnosis of lung cancer; the most obvious lymph node was sampled for standard of care testing and for research sampling, including Diff-Quik smears from each needle pass and fresh tissue samples. The contents of Diff-Quik smears were scraped for DNA extraction as previously described (6). To evaluate cytology smears as a potential source of DNA for sequencing with TSO500 (Illumina), we previously selected 27 cases with Diff Quik smears from a diagnostic EBUS TBNA procedure (6). Cases were selected either because they had SOC mutations (13 cases) or because there were multiple smears with a wide range of tumour content which would allow testing of the feasibility of TSO sequencing across a wide range of smear cellularities. We were able to sequence 50 smears using TSO 500. For this paper we did further detailed analysis on 20 of those patients from the original ethics approved study (45 smears) where there were at least 2 smears per patient, so as to study intra-procedural variation of TMB results.

Methods and data related to the microscopic estimation of tumour content on the Diff-Quik slide (involving estimating percent malignant cells to non-malignant cells, the approximate overall abundance of malignant cells and area of the glass slide covered by the smear), DNA extraction and sequencing by (TruSight Oncology 500 (TSO500 - Illumina)and the description of somatic mutations and TMB was previously reported (7, 8). The sequence data was processed and analysed using the TruSight Oncology 500 Local App version 2.0 (Illumina), which estimates TMB using eligible variants/effective panel size (coding region with >50x coverage). The vendor pipeline uses nonsynonymous and synonymous variants (single nucleotide variants and indels with allele frequency ≥ 5% and <90% that pass the inbuilt germline and COSMIC filters). Here we report on a subset of 20 patients with TSO500 sequencing derived from these Diff-Quik smears, with two to four smears per patient, giving a total of 45 smears for this analysis. To corroborate sequencing data for a particular case (Patient U), additional Whole Genome Sequencing (WGS) fresh tumour and matched normal was performed and mutations called previously described (9). cfDNA was extracted using the cfDNA extraction kit (Qiagen), and 30ng cfDNA was sequenced using the TSO500 cfDNA assay (Illumina) by the Australian Translational Genome Centre. Data was analysed using Dragen v2.1. Results of summary data are expressed as mean ± standard deviation.

## Results

**Supplemental Table 1** details the clinical information regarding the 20 patients, including tumour stage, treatment, standard of care biomarker results and length of survival, as well as the Diff-Quik smear microscopic evaluation of the 45 smears, TSO500 sequencing metrics and TMB scores. There were 9 cases of Adenocarcinoma, 7 Non Small Cell Carcinoma (NSCLC), 1 Small Cell Carcinoma, and 2 distant cancers metastatic to the lung. **Table 1** summarises TMB metrics. The meanTMB for all smears was 9.0± 5.5 mutations/Mb. Sixteen of the 20 patients had paired samples with minimal differences in TMB score (difference between smears mean ± standard deviation 1.3 ± 0.7 mutations/Mb) and 13 patients had concordance between smears in terms of all TMB scores in a given case being below or above a threshold of 10 mutations/Mb. Discordance between TMB scores was seen between paired smears in seven cases; for three of these cases the scores were considered similar (mean TMB difference 1.9 ± 1.0 mutations/Mb), but just happened to straddle the 10 mutations/Mb threshold, but for four cases (Patients C, I, T and U) the TMB scores were strikingly discrepant, with mean difference of 11.3 ± 2.7 mutations/Mb) (**Table 1**).

**Table 1:** Summary of TMB values defined by TSO500 from Diff Quik smears in the cohort.

Patient	Tumour Type	Tumour Mutation Burden (TMB) from each slide	TMB difference (absolute) between pairs of slides	Pairs with minimal difference in TMB	Pairs with concordant TMB	Pairs with discordant TMB	Average ± STD of the difference between samples
A	Adenocarcinoma	3.9		Y	Y		
		5.5	1.6				
		5.5					
		6.3	0.8				
B	Adenocarcinoma	18.8		Y	Y		
		21.9	3.1				
D	Adenocarcinoma	16.4		Y	Y		
		17.2	0.8				
F	Adenocarcinoma	4.7		Y	Y		
		6.3	1.6				
H	Adenocarcinoma	14.8		Y	Y		
		16.4	1.6				
J	Adenocarcinoma	0.8		Y	Y		
		1.6	0.8				
O	NSCLC	4.7	0	Y	Y		
		4.7	1.6				
		6.3	1.6				
P	NSCLC	7		Y	Y		
		7.8	0.8				
Q	NSCLC	11		Y	Y		
		12.5	1.5				
S	NSCLC	7		Y	Y		
		8.6	1.7				
X	SCLC	10.2		Y	Y		
		10.9	0.7				
Y	Breast	2.3	0	Y	Y		
		2.3	0.8				
		3.1	0.8				
Z	Melanoma	6.3		Y	Y		
		7	0.7				1.3 ± 0.7
E	Adenocarcinoma	9.4		Y		Y	
		10.2	0.8				
N	NSCLC	8.6	2.3	Y		Y	
		10.9	0.8				
		11.7	3.1				
R	NSCLC	8.6		Y		Y	
		10.9	2.3				1.86 ± 1.02
C	Adenocarcinoma	9.4				Y	
		19.5	10.1				
I	Adenocarcinoma	3.9				Y	
		18.8	14.9				
T	NSCLC	0.8				Y	
		12.5	11.7				
U	NSCLC	3.9				Y	
		12.5	8.6				11.33 ± 2.7

Green cells denote cases with significantly discrepant TMB scores; blue cells denote cases with TMB scores that were similar but still discrepant.

Uppercase letters are lists of alphabetical patient identifications.

*Discrepant TMB scores*: TMB was calculated by the vendor software based on the sequencing footprint with sufficient coverage, which was 1.28Mb of the genome for the samples in this cohort. We applied a threshold for TMB high of >10 mutations/Mb, which in this analysis equated to 13 or more variants that pass vendor filters.

Three cases were considered to have similar but still discrepant TMB values between paired smears (Patients E, N and R; **Table 1**; **Supplementary Table 1**). These paired samples differed by one to four mutations only, highlighting the subtle nature of classifying TMB as low versus high using a set threshold when using gene panels.

**Figure 1:**
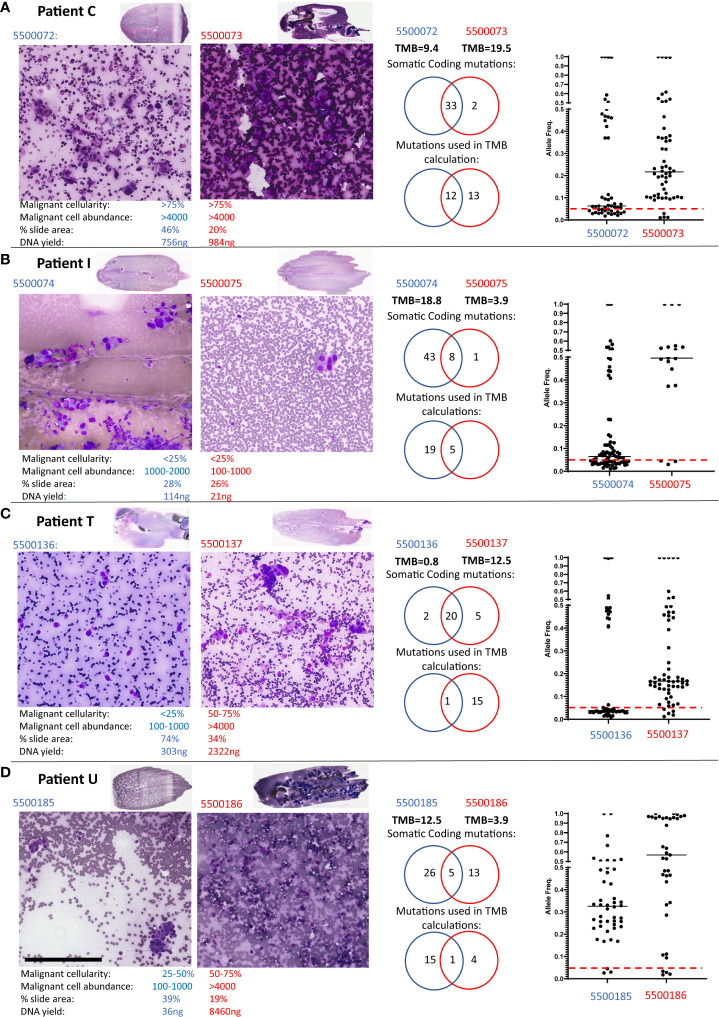
Patients with discordant TMB between paired smears. Low and high-power views of the paired Diff-Quik smears sequenced by TSO500 from Patient C **(A)**, Patient T **(B)**, Patient I **(C)** and Patient U **(D)**. Black bar in panel D represents 200μm. The paired Diff-Quik smear cytological evaluation involved estimation of the percentage malignant content of the smear, the malignant cell abundance (approximate number of malignant cells), the percentage area of the slide covered by smear and the DNA yield. Each panel also displays mutations called by the TSO500 using Local App version 2.0: a Venn diagram of the somatic coding mutations, a Venn diagram of the number of mutations used in the TMB calculation and a plot showing the allele frequency of somatic coding mutations in the two smears from each patient (the red line indicates an allele frequency of 5% used in the TMB calculation).

Four cases had significant differences in TMB scores: Case C and I (both Adenocarcinomas), and T and U (both NSCLC). **Figure 1** presents a detailed evaluation of paired smears used for each case, displaying cytology evaluation, the number of coding mutations detected, the number of mutations used to calculate the TMB, the overlap of mutations between smears and the allele frequencies of mutations in each smear. Data for Patients C, I and T indicate that differences in malignant cell content and DNA yield impact the sensitivity of detecting variants or the allele frequency of variants detected and hence discrepant TMB scores between paired smears:

Patient C (**Figure 1A**): cytology metrics were similar for both smears (>75% malignant cell content, high tumour cell abundance), and almost identical somatic mutations were called between smears. However, the majority of mutations were below the 5% allele frequency threshold in slide 5500072 resulting in a significant difference in TMB scores between smears. In retrospective analysis, slide 5500073 had large solid cell clusters making estimates of cell count difficult and hence under-estimating tumour content on that smear. A higher tumor content in sample 5500073 compared to 550072, agrees with the allele frequencies observed in the sequencing data.

Patient I (**Figure 1B**): cytology review indicated both smears were low malignant cellularity (<25%) yet slide 5500075 also had a low estimated malignant cell abundance (100-1000 cells versus 1000-2000 malignant cells in 5500074). These combined low tumour content metrics consequently led to both a low DNA yield and to less than the recommended DNA input (30ng) being used in TSO500 sequencing for smear 5500075. Sequencing sensitivity was therefore compromised, with few somatic coding mutations being detected (9 versus 51) and used in TMB scores (5 versus 24) compared to its paired slide (**Table 1**; **Supplementary Table 1**). Our previous study has reported a higher failure rate of sequencing metrics in samples with <25% malignant cells (7). These samples might allow detection of actionable mutations but need to be carefully considered for TMB estimation.

Patient T (**Figure 1C**): Here smear 5500136 was of lower malignant cellularity (<25% versus 50-75%) and abundance (100-1000 versus >4000) compared to its paired slide (5500137). Thus, while the somatic coding mutations detected were nearly identical, the variant allele frequency for most mutations in 5500136 was below the 5% threshold used to include variants in the TMB calculation.

**Figure 2:**
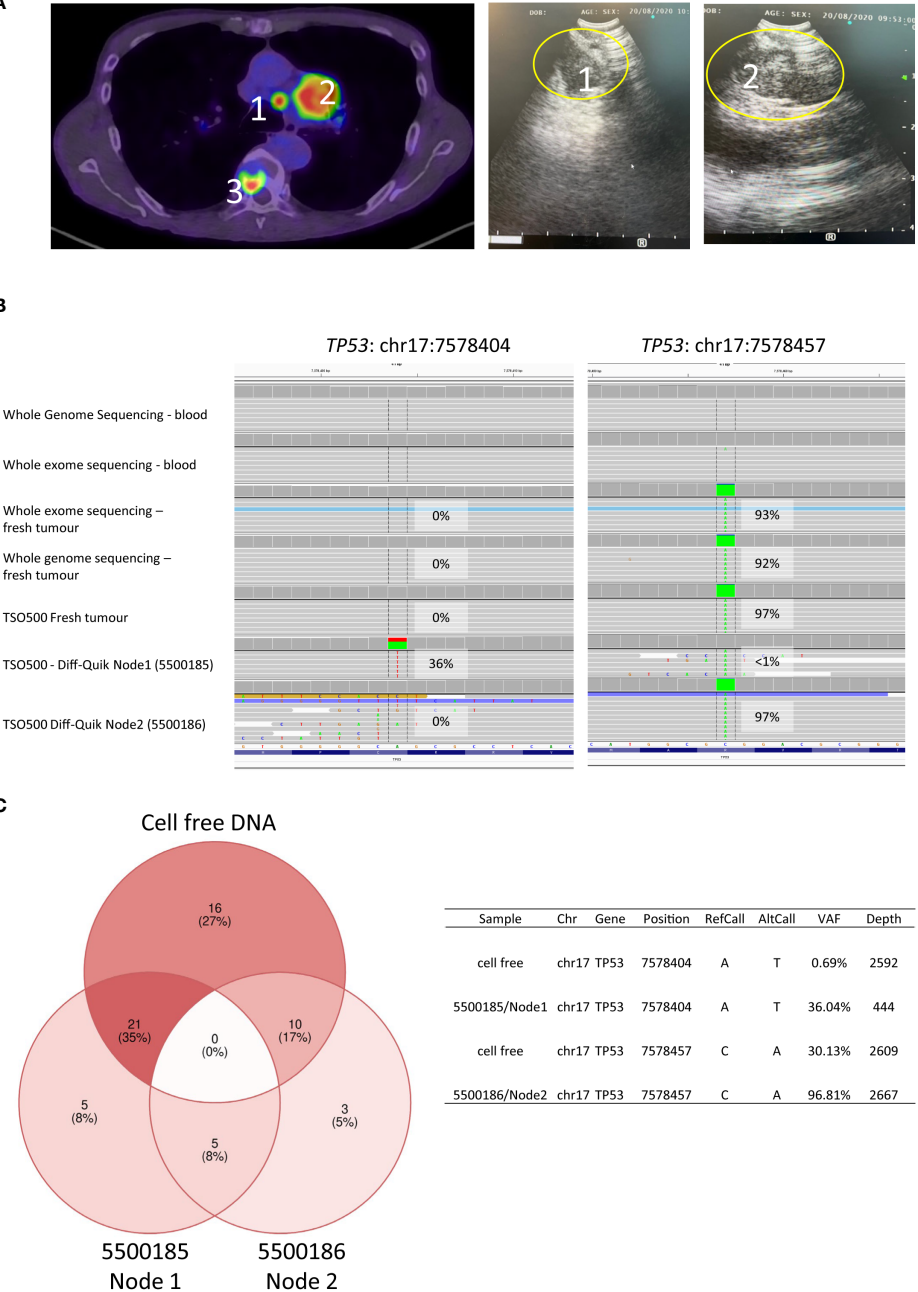
Intra-tumour heterogeneity in Patient U. **(A)** PET scan and EBUS clinical images: PET scan demonstrates strong avidity within 2 lymph nodes in the 4L station sampled during EBUS TBNA and shown by ultrasound images captured during bronchoscopic procedure. Additionally, the PET shows an avid site of spinal metastasis. **(B)** Images taken from Integrative Genomics Viewer (IGV) during manual review of somatic mutations detected in *TP53* positions chr17:7578404 and chr17:7578457. Tracks in IGV show respective variant position detected in each smears and other sequencing: whole genome sequencing (WGS) and whole exome sequencing (WES) of matched blood (germline) as well as WGS, WES and TSO500 sequencing from freshly collected EBUS TBNA aspirate of node 2; TSO500 sequencing of Diff-Quik smear from lymph nodes 1 and 2. Mutation chr17:7578404 was only detected in smear of lymph node 1 (smear 5500185) in 36% of reads; whereas mutation chr17:7578457 was detected in 92-97% of reads in all sequencing of the aspirate from lymph node 2. There is very low evidence of this mutation in reads (<1%) from lymph node 1 and so was not called by TSO500 Local App version 2.0. **(C)** A blood sample was also collected at the time of the bronchoscopy from which cell free DNA from plasma was sequenced using the cfTSO500 assay (Illumina). Venn diagram shows coding mutations detected in the Diff-Quik of nodes and cfDNA. Both *TP53* mutations noted above were detected at the variant allele frequencies shown in the table.

Patient U (**Figures 1D**; **2**): both the paired smears had reasonable malignant cell content (25-50% versus 50-75%; 100-1000 cells versus >4000 cells, 5500185 and 5500186, respectively). There was a difference in the number of mutations detected in each sample with little overlap of somatic coding mutations detected (**Supplementary Table 1**). The allele frequency for mutations in both smears were consistently >5%. The high number of somatic mutations unique to each smear (26 versus 13) resulted in highly discrepant TMB scores (12.5 versus 3.9). The different set of coding mutations in tumor protein p53 (*TP53)* and other key genes suggesting evidence of tumour heterogeneity. On closer examination of the clinical procedure it was revealed that two separate lymph nodes within the same lymph node station (4L) had been sampled (**Figure 2A**). At the time the clinician did not appreciate that there were two separate lymph nodes within the same lymph node station. Indeed, it would be somewhat difficult to specifically target one or the other of these lymph nodes with the EBUS TBNA needling technique given the limited access in this particular location (the left lower paratracheal location, 4L) which is commonly regarded as a more difficult site to aspirate. The smaller lymph node 1 (**Figure 2A**) corresponded to the smear (5500185) with high TMB (12.5), whereas the large lymph node 2 corresponded to the smear (5500186) with low TMB (3.9). Additional comprehensive sequencing data was available for this case, including whole genome (WGS) and whole exome (WES) sequencing of tumour and matched blood and TSO500 of a freshly collected EBUS TBNA aspirate sample. Analysis of germline variants detected in matched blood, smears and fresh EBUS material suggested all samples studied were from the same patient (not shown). *TP53* variant at position chr17:7578404 was detected only on the Diff-Quik material from lymph node 1, at an allele frequency of 36%. A different *TP53* variant (at position chr17:7578457) was detected in the Diff-Quik sample from lymph node 2 (5500186). It was also present in the fresh specimen by WGS, WES and TSO500 at 97% allele frequency (**Figures 2B, C**). Manual inspection of the reads (IGV, **Figure 2B**) showed very low evidence of this mutation in the tumour sample obtained from Node 1 (<1% of reads), hence it was not called. This data supports the idea that discrepant mutation calls between smears in this case were likely due to intra-tumour heterogeneity, with separate tumour clones colonizing separate lymph nodes. We also extracted cell free DNA from a blood plasma sample obtained from this patient at the time of their bronchoscopy procedure. Most mutations detected in nodal smears were also detected in cfDNA (**Figure 2C**); this included both *TP53* mutations that were detected in the cfDNA at variant allele frequencies reflecting the allele frequency and size of the LN nodules: variant at position chr17:7578404 detected at VAF of 0.69% and variant at position chr17:7578457 detected at 30.13%. Blood TMB from cfDNA of this patient was 24mut/Mb. There is no consensus on the threshold of blood TMB associated with response to immunotherapy: studies have reported thresholds of 16 mutation/Mb or 20 mutations/Mb (10, 11).

## Discussion

In this brief report, we further evaluate panel sequencing data of EBUS TBNA samples collected in a cohort of cases previously reported (7, 8) to explore TMB reporting. The study is important because EBUS TBNA samples are commonly collected for diagnosing lung cancer, and Diff Quik smears represent an under-utilized tissue resource for molecular diagnostics. To the best of our knowledge this is the first detailed report of TMB on cytology smears. The study was unique for 2 reasons. Firstly, we utilized a unique sampling strategy whereby malignant lymph nodes had repeat samples taken, thereby allowing for analysis of the reproducibility of the test. We found that repeat samples of individual lymph node sites generally have minimal variation in the detection of actionable mutations (7). Multiple smears from the same node enabled us to demonstrate the impact of tumour content on TMB analysis. In 20 patients with multiple slides, 16 paired samples had similar TMB, suggesting high reproducibility of the Diff-Quik smears. Secondly, we have demonstrated a new way Diff Quik smear material can be used, namely for large panel sequencing to allow TMB testing, a significant clinical feasibility.

However, discrepant results do occur, as suggested in published results comparing fresh tissue of matched primary and lymph node sites (5, 12), and this is clinically important given that TMB could support a decision regarding treatment. Here we highlight parameters that significantly influence TMB estimation and the subsequent classification of a tumor as TMB high or low. As expected, and inferred by others, the key factors being tumour content of the sample, which impacts DNA yield and input for sequencing and intra-tumour heterogeneity; and how subtle variation in somatic mutation calling can impact classification.

Tumour content of a sample is the combined sum of the estimated malignant cellularity (or tumour content), the abundance of malignant cells and the size of the specimen, in this case a cellular smear. Collectively these factors all affect the yield of DNA from a sample and the likely sensitivity for detecting somatic (tumour specific) mutations in malignant cells relative to the proportion of contaminating normal cells (respiratory epithelium, stroma and immune cells). In the recent Checkmate227 trial of nivolumab plus ipilimumab versus chemotherapy, TMB was assessable in 58% of patients, with available tissue quantity and quality being the limiting factors in this assessment (13). Here we show with a paired slide analysis, that while TMB can be assessed off almost all cytology smears with successful sequencing, the robustness of TMB calculation is impacted by low cellularity, abundance and/or smear size. Most previous studies assessing TMB used fresh or FFPE material, here we are the first to show that cytology slides (smears) could be used for sequencing and TMB estimation, with the advantage that the slides provide a visual estimation of tumour content to support the interpretation of the sequencing data. Of the four cases with significantly discrepant TMB scores, two were clearly caused by combined low cellularity (<25%) and low abundance (<1000 cells) metrics. We previously reported that smears that failed sequencing coverage metrics had <25% tumour content (7), suggesting this is a useful threshold for selecting smears for sequencing. Nevertheless, it is worth noting these metrics are not always associated with discrepant TMB, with a further four smears (from patients A, H, O, S) scored with <25% cellularity yielding concordant TMB with paired smears of higher tumour content (**Table 1**). Further, estimating these cytology metrics can be challenging depending on the smear quality and content, as noted for smears in Patient C (cell clusters), and further the scale of tumour content within the ‘<25%’ quartile may also be significant (effectively being between 1% to 24%) and impact the robustness of sequencing. This study shows the advantages of digital scanning of smears prior to extracting DNA to enable retrospective review of images and sequencing data together.

This study exemplifies why using smear samples for genomics is useful as these factors can be quickly estimated based on the what-you-see-is-what-you-get nature of the smear. Our analysis indicated that discrepancies in TMB arise from both genuine tumour heterogeneity but also from sample inadequacy due to instances of low tumour content.

Two recent papers demonstrate the great potential of cytologic specimens for TMB estimation (analysed from cell blocks, not smears). Pepe et al. (14)reported 8 pairs of histologic and cell block samples from primary tumours (6) and lymph nodes (2). Six of 8 cell blocks were successfully sequenced; factors used in TMB assessment were similar between the histologic and cell block samples including median total reads, median average reads per amplicon, and median uniformity of amplicon coverage. They concluded further prospective study was required on the use of cell blocks (and indeed smears as well) given the lesser impacts of formalin on these samples compared to histologic samples. Similarly Alborelli (15) reported matched cell block (Formalin fixed paraffin embedded, FFPE) specimens from surgical resection samples, and cytology smear samples made from those resection samples in 12 patients. Mutations used for TMB calculation were concordantly detected in matched histological and cytological samples. Again cytology specimens were effective, and had far fewer discordant variants which were mainly unique to FFPE samples (34/40 discordant variants). Again the authors considered these due to formalin fixation artifacts in the histologic specimens. Regarding changing the VAF frequency (for reporting), FFPE samples showed 2 out of 12 patients classified as “TMB-high” at VAF cutoff of 5%, but “TMB-low” at 10%. This change of VAF threshold did not affect cytology specimens.

Clinicians should also be aware that even slight differences in lymph node site selection could give rise to variable mutations and discrepant TMB results caused by intra-tumour heterogeneity. Here, because of the inadvertent sampling of two separate nodes within the same lymph node station by EBUS TBNA, we observed evidence that separate lymph nodes were likely colonized by separate neoplastic clones. When performing EBUS TBNA sampling, staging guidelines recommend that where a particular lymph node station has abnormal nodes on CT or PET, then several nodes within that station should be sampled (16). Case U showed molecular heterogeneity between nodes even within one node station and so this adds potential value to this recommendation. The fresh tissue sample (into which all aspirates from the 1 station were placed), appeared to have only material from node 2, based on analysis of the TP53 mutation. Presumably because node 1 was smaller and likely difficult to access very little aspirate from that node could be obtained for the fresh research sample, even though a good quality Diff-Quik smear could be made. Hence the majority of the fresh sample was from node 2 with lower TMB. Recent detailed multi-regional and longitudinal sampling and sequencing in the TRACERx consortium elegantly illustrated the scale of clonal evolution within patient primary lung cancers and subsequent metastases, demonstrating that 31% of cases studied showed evidence of polyclonal dissemination, in which multiple clones within a primary tumour seeded metastases (4, 17). Intra-tumour heterogeneity is an important clinical issue to understand treatment response/resistance and in the context of TMB as to whether to use immunotherapy or not. Where a patient has only one or perhaps two stations involved, the likelihood of significant tumour heterogeneity between those lymph node stations may not be significant however clinicians should be aware that where multiple lymph node stations are involved then this heterogeneity could well exist. It would be difficult to recommend sequencing on every aspirate from within one station. However it would be reasonable for multisite analysis of TMB to be performed using EBUS TBNA aspirates from different easily accessible lymph nodes from different stations, even if they were in an N2 or N3 category, as suggested previously (5). Diff-Quik smears could make an ideal way to easily label the site of the aspirate sample to allow this multisite sequencing. We showed that the use of the archived digital smear image together with sequencing results could guide clarification of discordant TMB results and confirm the most likely real estimation or if nodal heterogeneity exists.

Future studies are needed to resolve questions regarding a multisite node sequencing analysis protocol such as cost effectiveness and frequency of change in management. Such studies should explore the selection of lymph nodes for sampling by EBUS TBNA, including lymph nodes further geographically separated from the primary tumour compared to those which are closer. The work should also consider the value of sequencing cell free DNA from liquid biopsy which has the potential to capture the scale of intra-tumour heterogeneity in one sample, but which may also suffer from sample inadequacy or intra-tumoral heterogeneity of blood perfusion.

In summary, Diff-Quik smears are an excellent source of material for genomic testing. They enable quick estimation of tumour content of the slide and the selection of the best smear(s) for genomic sequencing. It is important to review the clinical procedural notes, the cytology metrics together with the sequencing data during interpretation and reporting of mutations and TMB classification. Analysis of multiple smears from different lymph nodes and/or cfDNA sequencing could help assess the existence of intra-tumour heterogeneity that may affect response or resistance to therapy.

## Data availability statement

The original contributions presented in the study are included in the article/**Supplementary Material**, further inquiries can be directed to the corresponding author.

## Ethics statement

The studies involving humans were approved by Metro North Hospital Health Service Human Research Ethics Committee ((HREC/17/QRBW/301), The University of Queensland (2005000785) and QIMR Berghofer (P2404). The studies were conducted in accordance with the local legislation and institutional requirements. The participants provided their written informed consent to participate in this study.

## Author contributions

DFi: Conceptualization, Data curation, Formal Analysis, Funding acquisition, Investigation, Methodology, Resources, Supervision, Visualization, Writing – original draft, Writing – review & editing. AD: Data curation, Formal Analysis, Investigation, Methodology, Resources, Writing – review & editing. MS: Formal Analysis, Methodology, Writing – review & editing. LN: Formal Analysis, Methodology, Writing – review & editing. VL: Data curation, Formal Analysis, Software, Validation, Writing – review & editing. HC: Data curation, Formal Analysis, Methodology, Validation, Writing – review & editing. DFa: Formal Analysis, Investigation, Validation, Writing – review & editing. A-MP: Data curation, Formal Analysis, Software, Validation, Writing – review & editing. SK: Data curation, Formal Analysis, Methodology, Software, Validation, Writing – review & editing. KF: Data curation, Methodology, Software, Writing – review & editing. FB: Investigation, Methodology, Writing – review & editing. MB: Writing – review & editing, Investigation, Methodology. CP: Investigation, Writing – review & editing. JS: Investigation, Writing – review & editing. KR: Data curation, Investigation, Writing – review & editing. AH: Investigation, Methodology, Writing – review & editing. SS: Investigation, Methodology, Writing – review & editing. JP: Data curation, Formal Analysis, Investigation, Methodology, Software, Validation, Writing – review & editing. NW: Data curation, Formal Analysis, Investigation, Methodology, Software, Validation, Writing – review & editing. SL: Conceptualization, Resources, Supervision, Writing – review & editing. GH: Data curation, Formal Analysis, Software, Validation, Writing – review & editing. PS: Conceptualization, Data curation, Funding acquisition, Investigation, Methodology, Project administration, Resources, Supervision, Validation, Visualization, Writing – review & editing. KN: Conceptualization, Data curation, Formal Analysis, Methodology, Resources, Software, Validation, Visualization, Writing – review & editing.
